# Primary undifferentiated pleomorphic sarcoma of the stomach mimicking a gastrointestinal stromal tumor: a case report and literature review

**DOI:** 10.3389/fonc.2026.1777324

**Published:** 2026-04-27

**Authors:** Jun Mao, Xiaonan Yin, Zhaolun Cai, Chaoyong Shen, Bo Zhang

**Affiliations:** 1 Gastric Cancer Center, West China Hospital, Sichuan University, Chengdu, China; 2 Department of General Surgery, West China Hospital, Sichuan University, Chengdu, China

**Keywords:** case report, gastrointestinal stromal tumor, soft tissue sarcoma, stomach, undifferentiated pleomorphic sarcoma

## Abstract

**Introduction:**

Undifferentiated pleomorphic sarcoma (UPS) is a rare and aggressive malignant soft tissue sarcoma. While most commonly arising in the extremities and trunk, primary occurrence in the stomach is exceedingly rare. Due to its rarity and nonspecific presentation, preoperative diagnosis is challenging and often established postoperatively through detailed histopathological and molecular analysis.

**Case presentation:**

We report the case of a 71-year-old female presenting with a gastric mass and anorexia, who was preoperatively misdiagnosed with gastrointestinal stromal tumor(GIST) based on clinical and imaging characteristics. Due to the massive tumor burden and anatomical complexity, the patient underwent an R0 resection combined with splenectomy. The final diagnosis of primary gastric UPS was established through an exclusionary process involving comprehensive histopathological, immunohistochemical (including negativity for CD117, CD34, DOG-1), and molecular analyses to rule out other tumors with specific lines of differentiation. Despite adjuvant doxorubicin chemotherapy, follow-up imaging revealed tumor recurrence two months postoperatively.

**Conclusion:**

This case underscores the highly aggressive biological behavior and dismal prognosis associated with primary gastric UPS. The rapid recurrence following multimodal therapy highlights the critical importance of considering UPS in the differential diagnosis of gastric soft tissue masses and emphasizes the urgent need for more effective, comprehensive therapeutic strategies.

## 1 Introduction

Undifferentiated pleomorphic sarcoma(UPS) is a rare malignant soft tissue sarcoma with uncertain differentiation, previously known as malignant fibrous histiocytoma (MFH) (1). Currently, epidemiological data on UPS remain limited, with an annual incidence of approximately 0.88 per 100,000 individuals (2). UPS most commonly occurs in the extremities and trunk, and often metastasizes to the lungs, lymph nodes, liver, and bone, whereas primary gastric cases are exceedingly rare (3).

The clinical presentation of UPS is nonspecific, often manifesting as a painless soft tissue mass. As the most common mesenchymal tumor of the digestive tract (4), gastrointestinal stromal tumor(GIST) shares similar clinical features and imaging characteristics on computed tomography (CT) and magnetic resonance imaging (MRI), making it difficult to distinguish from UPS. Histopathologically, UPS is characterized by pleomorphic or spindle−shaped tumor cells, and diagnosis requires exclusion of other neoplasms with specific lines of differentiation (3).

In accordance with the CAse REport (CARE) guidelines (5), we present a case of primary gastric UPS that was initially misdiagnosed as GIST. The patient underwent R0 surgical resection followed by adjuvant chemotherapy, yet experienced recurrence two months postoperatively. This case highlights the importance of considering UPS in the differential diagnosis of gastric soft tissue masses and underscores the need for comprehensive therapeutic strategies.

## 2 Case presentation

A 71-year-old female presented to our institution on January 26, 2024, with a 5-year history of a gastric mass and more than one month of anorexia. Her past medical history was notable for severe pancreatitis five years prior and a cholecystectomy four years prior. Upon admission, the patient’s vital signs were stable (temperature, 36.7 °C; blood pressure, 132/78 mmHg; heart rate, 72 bpm; respiratory rate, 16 breaths/min). Abdominal examination revealed a soft, non-tender abdomen with no palpable mass. Bowel sounds were normal. Routine laboratory investigations showed no significant abnormalities. Furthermore, she had no family history of malignancy or genetic disorders, no prior interventions for the gastric tumor, and an unremarkable psychosocial history.

**Figure 1:**
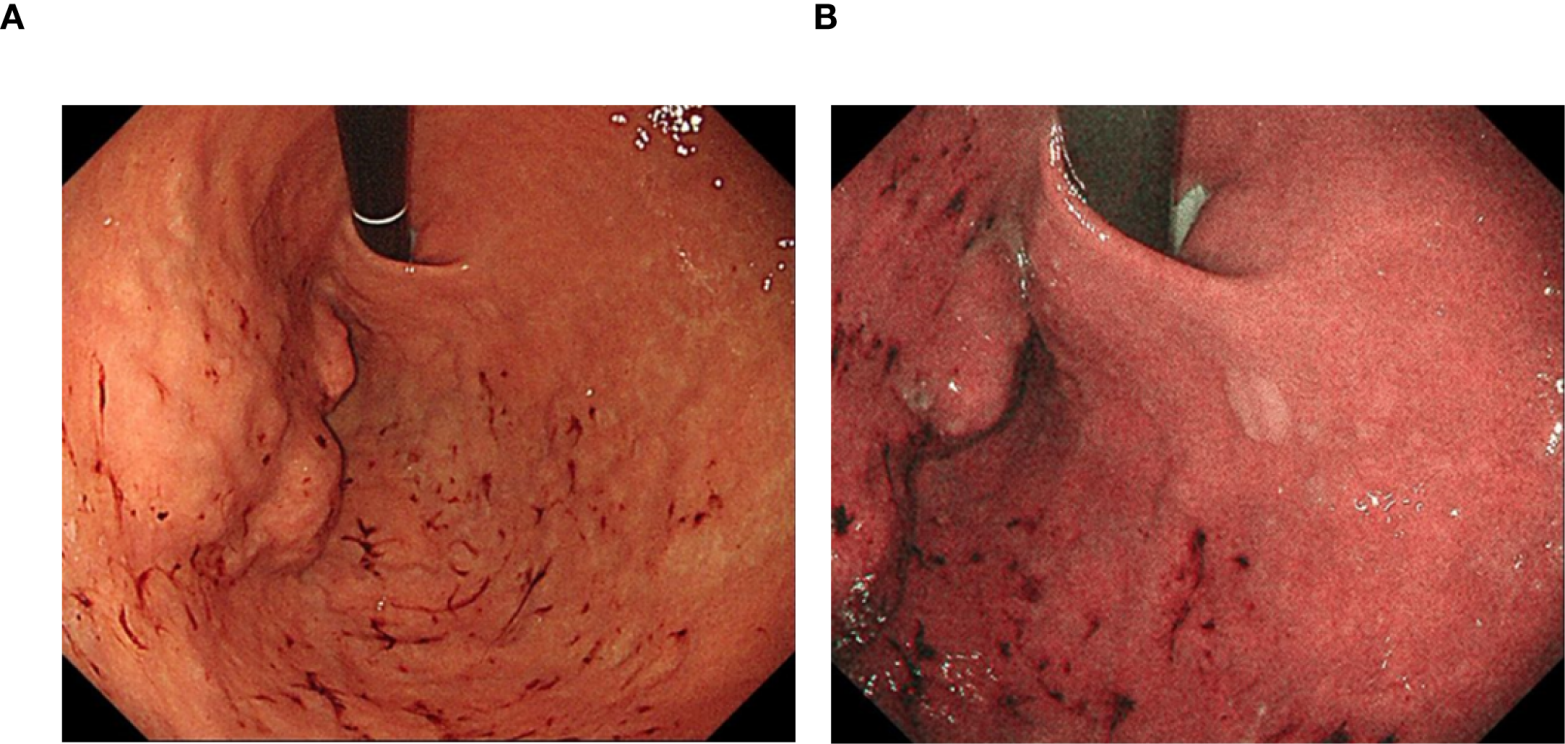
Endoscopic appearance of the gastric mass **(A)** with hemorrhagic spots and polyps **(A, B)**.

**Figure 2:**
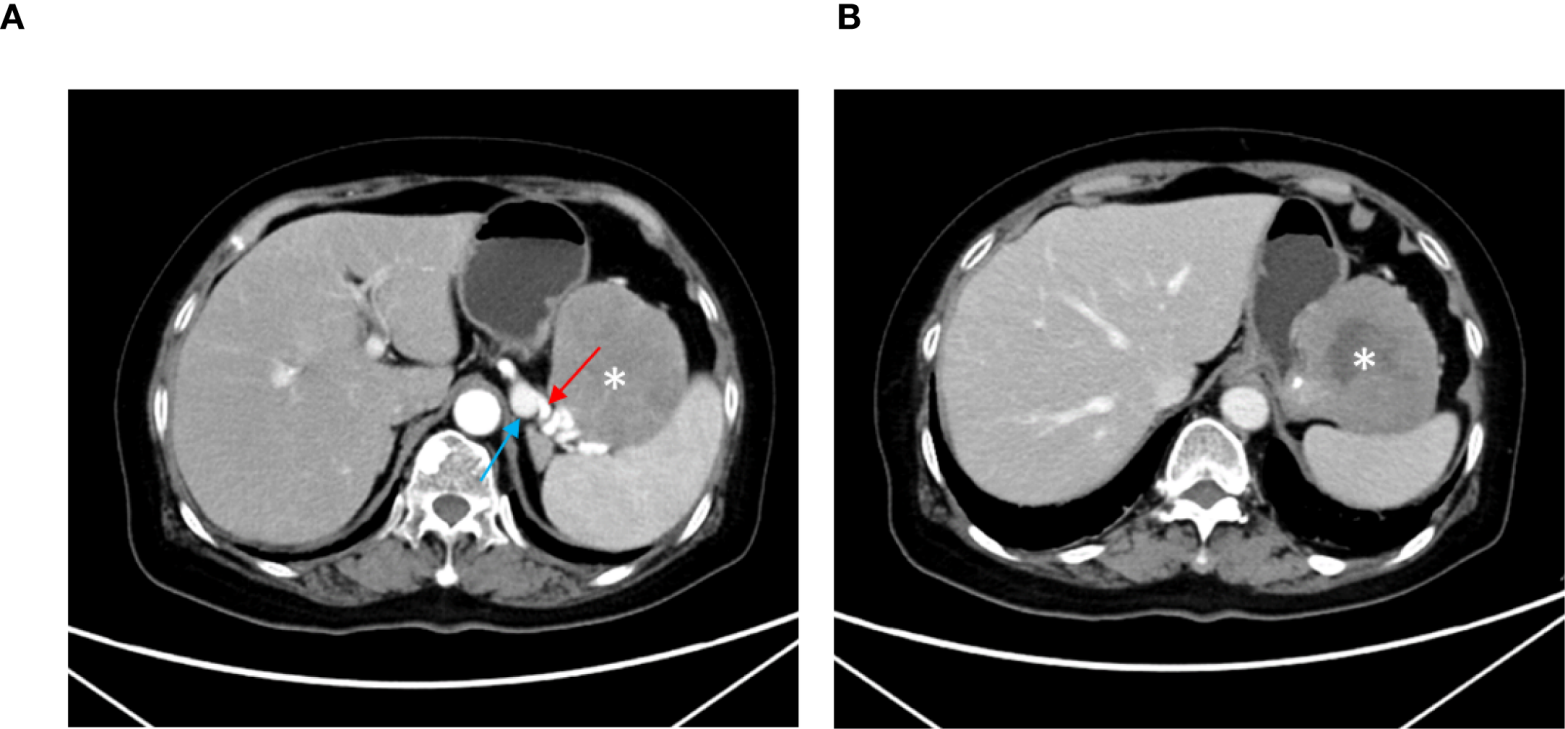
Contrast-enhanced abdominal CT scan of the gastric mass. **(A)** The exophytic mass at the fundus and the greater curvature (asterisk), with indistinct boundaries separating it from the splenic artery (red arrow) and splenic vein (blue arrow); **(B)** The mass contained sparse high-density foci and areas of liquefactive necrosis (asterisk) cm.

10 days before admission, the gastroscopy revealed scattered old hemorrhagic spots in the gastric fundus, a mucosal protuberance measuring approximately 5.0 cm, and scattered flat polyps (0.2–0.5 cm in diameter) with smooth surfaces (**Figure 1**). Because it was uncertain whether the lesion represented a submucosal tumor or external compression, and given the diagnostic uncertainty, the potential inability of biopsy to obtain representative tissue, and the risk of damaging normal structures, no biopsy was performed at that time. Contrast-enhanced abdominal CT performed on January 20, 2024, demonstrated a soft-tissue density mass measuring approximately 7.7 × 7.4 cm, located at the gastric fundus and the greater curvature, exhibiting exophytic growth (**Figures 2A, B**). The mass contained sparse high-density foci and areas of liquefactive necrosis (**Figure 2B**). It was intimately adherent to the medial margin of the spleen, with indistinct boundaries separating it from the splenic artery and vein (**Figure 2A**). The gallbladder was absent. Chest CT demonstrated a 0.3 cm nodule in the left lowerlobe (considered most likely inflammatory) (**Supplementary Figure 1**), bronchiectasis in the lower lobes bilaterally, minimal bilateral chronic inflammatory changes, and slight aortic wall calcification. No other significant findings were identified.

The patient was initially diagnosed with a GIST. Following a consultation regarding the patient’s condition and surgical risks, an open gastric tumor resection was performed on January 29, 2024. A midline upper abdominal incision of approximately 10 cm was made, and the abdomen was entered layer by layer. After opening the gastrocolic ligament, the gastric fundus and body, along with the tumor, were exposed. Intraoperative exploration revealed a massive tumor measuring approximately 10 × 12 cm at the gastric fundus and greater curvature, originating from the submucosal layer and exhibiting invasive exophytic growth. Then the gastric wall was transected 2 cm from the tumor margin. The left side of the tumor was intimately adherent to the splenic hilum, encasing the splenic vessels and pedicle, rendering separation impossible. Owing to the tumor’s hypervascularity and dense adhesions secondary to prior pancreatitis, dissection proved exceedingly difficult. Therefore, the decision was made to extend the incision leftward and perform an en bloc splenectomy to achieve complete resection. The gastric wall was closed with full-thickness 4–0 absorbable sutures, and the seromuscular layer was reinforced with imbricating sutures. Ultimately, the patient underwent curative resection of gastric GIST, adhesiolysis, and splenectomy. Intraoperative frozen section analysis suggested a gastric spindle cell tumor, with no evidence of tumor involvement at the gastric resection margins. Three days later, permanent-section pathological examination of the frozen tissue confirmed the same findings.

**Figure 3:**
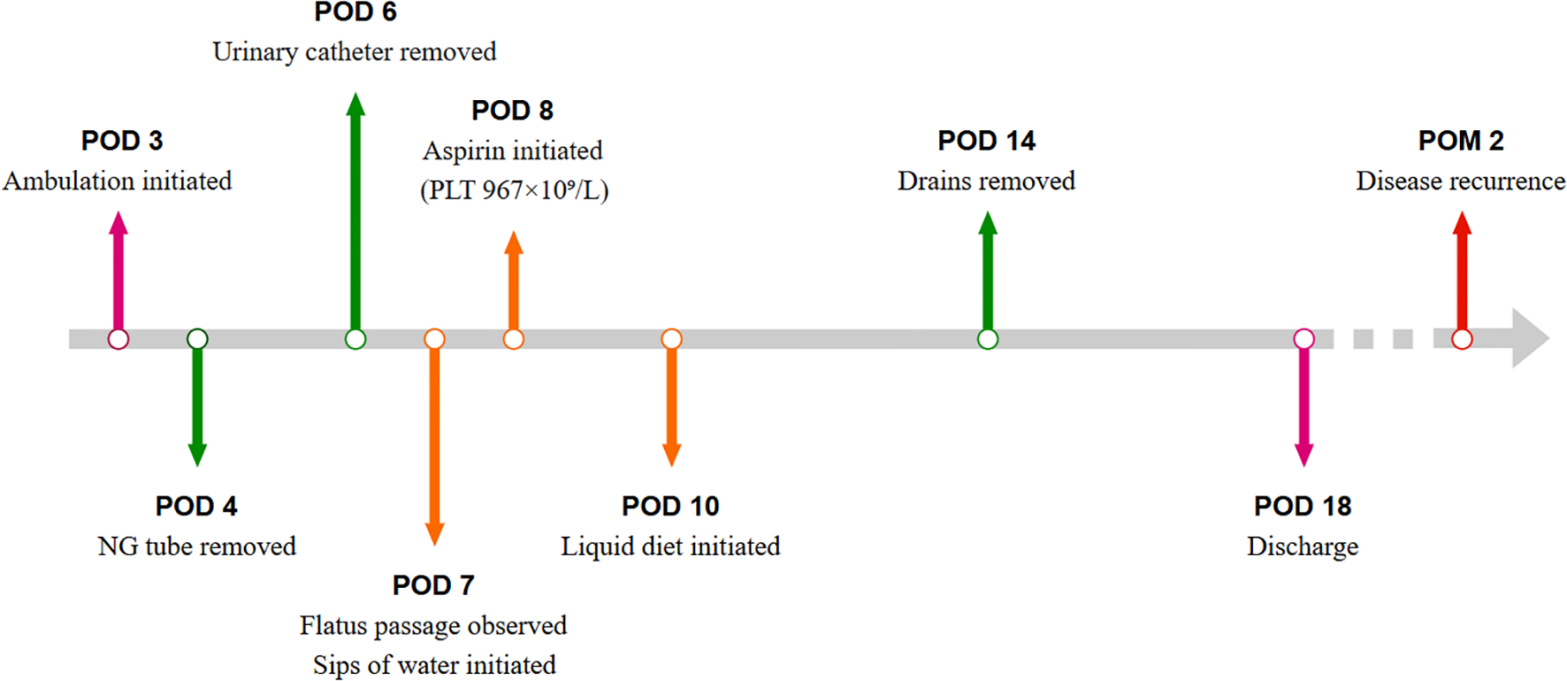
Postoperative clinical course timeline. POD, postoperative day; POM, postoperative month; NG, nasogastric; PLT, platelet count.

The operation lasted 330 minutes, with an estimated blood loss of 1,400 mL. Intraoperative transfusion included 600 mL of fresh frozen plasma and 9.5 units of packed red blood cells, with a total fluid administration of 3,900 mL. Two drainage tubes were placed adjacent to the greater curvature of the stomach. The nasogastric tube was removed on postoperative day (POD) 4. Flatus passage was observed on POD 7, at which point limited water intake was initiated. Blood tests on POD 8 revealed a platelet count of 967 × 10^9^/L, attributing this to secondary thrombocytosis following splenectomy, and oral aspirin therapy was administered. A liquid diet was initiated on POD 10, and the patient was discharged on POD 18. The timeline of key postoperative clinical events is shown in **Figure 3**.

**Figure 4:**
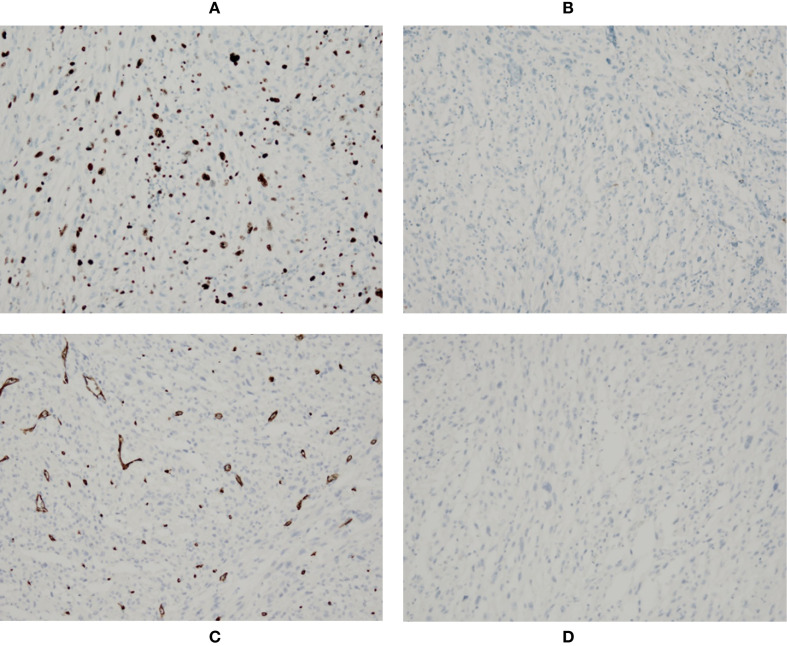
Immunohistochemistry stains showed that the tumor cells were positive for Ki-67 **(A)**, and negative for CD117 **(B)**, CD34 **(C)** and DOG-1 **(D)**.

Histopathological examination of the postoperative specimen revealed a pleomorphic and spindle cell tumor in the stomach, with focal calcification and the presence of tumor giant cells. The mitotic count exceeded 5 per 5 mm^2^ (**Supplementary Figure 2**). Immunohistochemical staining demonstrated that the tumor cells were positive for pan-cytokeratin, H3K27Me3, SDHB, INI1, and BRG1, with partial positivity for CD68 (PG-M1), S100, p16, and Ki-67 (approximately 50%) (**Figure 4A**), and weak positivity for desmin, epithelial membrane antigen (EMA), and CDK4. The neoplastic cells were negative for CD117 (**Figure 4B**), CD34 (**Figure 4C**), DOG-1 (**Figure 4D**), smooth muscle actin (SMA), STAT6, TLE1, SOX10, HMB-45, ALK, ROS1, TRK (Pan), CD21, CD23, SSTR2, synaptophysin (Syn), CD56, MUC4, Wilms tumor 1 (WT1), GATA3, calretinin (CR), cytokeratin 5/6 (CK5/6), cytokeratin 8/18 (CK8/18), CDX2, NUT, and NKX2.2. Fluorescence *in situ* hybridization (FISH) analysis did not detect *MDM2* gene amplification. Molecular genetic testing identified a *TP53* c.517G>A (p.Val173Met) missense mutation, resulting in the substitution of valine with methionine at amino acid position 173. The tumor mutation burden (TMB) was low, and microsatellite instability (MSI) testing confirmed the tumor to be microsatellite stable (MSS). Based on the integration of these findings, a final diagnosis of undifferentiated pleomorphic sarcoma was established. Accordingly, the patient received adjuvant chemotherapy with doxorubicin postoperatively. During treatment, the patient demonstrated good adherence and reported no significant discomfort such as nausea, vomiting, decreased appetite, or abdominal pain. No severe adverse events occurred. However, the follow-up abdominal CT at two months after surgery indicated tumor recurrence.

## 3 Discussion

We present a rare case of primary UPS of the stomach in a 71-year-old female. The nonspecificity of the clinical and imaging presentations led to a preoperative misdiagnosis of GIST. Owing to the extensive tumor burden and anatomical complexity, an R0 resection with concomitant splenectomy was performed. However, the patient suffered early recurrence despite postoperative adjuvant chemotherapy. This case exemplifies the aggressive biological behavior and dismal prognosis associated with gastric UPS, highlighting the significant challenges that remain in its diagnosis and treatment.

A systematic search was conducted in the PubMed database using the keywords “undifferentiated pleomorphic sarcoma” and “malignant fibrous histiocytoma” up to December 2025. A total of 12 case reports of gastric UPS, including the current case, were included (**Table 1**) (6–14). The age of patients ranged from 37 to 82 years, with a median age of 69, predominantly affecting males. Tumors were most commonly located in the body, fundus, and antrum of the stomach, particularly on the greater curvature, and most cases presented with large tumor sizes (diameter > 5 cm). Clinical manifestations mainly consisted of nonspecific gastrointestinal symptoms. All patients underwent surgical resection, with only two cases receiving adjuvant chemotherapy. Follow-up results indicated a poor prognosis for gastric UPS, with most cases experiencing recurrence or metastasis shortly after surgery. Approximately, half of the patients died within two years postoperatively, with only a minority surviving beyond five years. This highly aggressive biological behavior is not unique to gastric UPS. As reported in the literature, UPS arising from other anatomical sites such as liver (15), retroperitoneum (16), and mediastinum (17) similarly demonstrates rapid progression and a poor prognosis.

**Table 1:** Clinical characteristics of reported cases of primary gastric UPS. post-op, postoperative.

No.	First author	Year	Age (years)	Gender	Clinical presentation	Site	Tumor size	Treatment	Outcome
1	Shibuya (6)	1985	60	Male	Hematemesis and melena	Gastric antrum at the greater curvature	4.5 × 4 cm	Total gastrectomy	Death at 3 months post-op
2	Wright (7)	1988	42	Male	Bleeding	Gastric cardia	5 cm	Partial gastrectomy and chemotherapy	Death at 17 months post-op
3	Rathakrishnan (8)	1989	51	Male	Epigastric pain, loss of appetite, weight loss	Extending along the lesser curvature to the gastric antrum	Not specified	Feeding jejunostomy	Death within weeks
4	Wada (9)	1998	78	Male	Epigastralgia and weight loss	Posterior wall of the lower gastric corpus	4.5 × 5.5 cm	Partial gastrectomy	Alive at 24 months post-op
5	Wada (9)	1998	77	Male	Epigastralgia	Anterior wall near the greater curvature of the antrum	4 × 4 cm	Partial gastrectomy	Death at 48 months post-op
6	Wiersema (10)	1998	37	Female	Worsening anemia	Posterior gastric wall along the lesser curvature	5 cm	En bloc resection (stomach, transverse colon, spleen and pancreatic tail)	Alive at 38 months post-op
7	Agaimy (11)	2007	79	Male	Non-specific large ulcer	Gastric fundus	8 × 7.5 × 5 cm	Total gastrectomy	Death due to pulmonary embolism at 2 weeks post-op
8	Agaimy (11)	2007	68	Female	Weight loss, abdominal mass	Greater curvature of the stomach	12 × 9 × 6 cm	Two-thirds gastrectomy (Billroth II)	Alive at 6 months post-op
9	Kabashima (12)	2017	82	Male	Fever, general fatigue, shaking chill	Upper part of the greater curvature	8 × 5 × 3.5 cm	Partial gastrectomy	Alive at 1 month post-op
10	Oguri (13)	2018	70	Male	Abdominal fullness	Upper stomach	14 × 12 × 10 cm	Total gastrectomy, resection of small intestinal metastases	Recurrence at 6 months post-op; alive for >7 years
11	Iwakawa (14)	2023	67	Female	Upper abdominal pain	Greater curvature of the fundus	8.5 × 8.0 × 6.5 cm	Total gastrectomy, splenectomy, partial resection of diaphragm and liver	Peritoneal dissemination at 8 months post-op; death at 10 months post-op
12	This case	2024	71	Female	Gastric mass and anorexia	Gastric fundus	10 × 12 cm	R0 resection and chemotherapy	Recurrence at 2 months post-op

While CT serves as the preferred imaging modality for evaluating gastric UPS (18), a definitive diagnosis relies on histopathological and molecular examination of the tumor tissue to exclude other tumors. In this case, a GIST was initially suspected based on imaging features and clinical experience. However, immunohistochemical analysis revealed negative staining for CD117, CD34, and DOG-1, which are typically expressed positively in GIST (4). Furthermore, liposarcoma typically features adipocytic differentiation often accompanied by *MDM2* gene amplification (1, 19), and was also excluded due to only weak positivity for CDK4 (20); leiomyosarcoma usually presents with fascicular or spindle cell morphology and positive staining for SMA and desmin (21); and malignant melanotic schwannoma is characterized by S100, SOX10 and HMB45 expression, frequently associated with inactivating mutations in the *PRKAR1A* gene (1); TLE1 was negative, EMA showed only weak positivity, and no *SS18* gene rearrangement was detected, thus synovial sarcoma was effectively ruled out (22); STAT6 negativity argues against the diagnosis of solitary fibrous tumor (23). Through comprehensive evaluation, these specific sarcoma subtypes were successfully excluded in this patient.

Notably, the expression of pan-cytokeratin and weak positivity for EMA in the present case are atypical for UPS, initially raising the differential diagnosis of undifferentiated or sarcomatoid carcinoma. However, the pan-cytokeratin staining was neither diffuse nor intense, and EMA was only weakly positive. Furthermore, a broad panel of epithelial-specific markers yielded negative results: negativity for CK8/18 and CDX2 effectively excluded an adenocarcinoma (24); GATA3 negativity argued against urothelial or mammary origins (25); and the lack of WT1 and calretinin (CR) expression ruled out mesothelioma (26); The absence of CK5/6 expression further diminished the likelihood of a squamous cell carcinoma component (27). Collectively, the complete lack of staining for all lineage-specific epithelial markers, in conjunction with the absence of morphological features indicative of glandular, squamous, or transitional cell differentiation, effectively excluded the possibility of an epithelial malignancy, including sarcomatoid carcinoma.

Similar phenomena have been documented in previous studies. Lucas et al. reported that 17% of sarcomas may express pan-cytokeratin (28), and Kushitani et al. likewise identified rare instances of cytokeratin expression in sarcomas (29). In these cases, the pattern of keratin expression is distinctly different from the diffuse, intense staining typically seen in carcinomas. Bahrami et al. emphasized that to avoid misdiagnosing sarcomas with aberrant keratin expression as carcinomas, it is imperative to employ an expansive immunohistochemical panel that includes both expected positive and negative markers (30). In this patient, the retention of INI1, BRG1 and H3K27Me3 (31–33), coupled with the negativity of multiple epithelial markers, strongly supports a sarcomatous lineage over a carcinomatous one. Consequently, these findings were interpreted as aberrant, non-specific epithelial marker expression within a UPS.

Currently, there is no standardized treatment protocol specifically for gastric UPS. According to general clinical guidelines for soft tissue sarcomas, surgical resection remains the cornerstone of treatment (18, 34). For resectable cases, neoadjuvant doxorubicin-based chemotherapy may be considered to achieve tumor downstaging and improve R0 resection rates, followed by postoperative adjuvant chemotherapy using the same regimen. For unresectable or metastatic cases, systemic therapy forms the therapeutic backbone—typically utilizing doxorubicin-based combinations—while palliative surgery or radiotherapy may be employed for local symptom control (34).

In addition, a *TP53* gene mutation was detected in our patient. A study involving 143 patients with soft tissue sarcoma reported that 43.1% of UPS patients harbor partial or complete deletions of the *TP53* gene, while 32.1% exhibit sequence mutations (35). Consequently, restoring the function of the p14ARF-MDM2-p53 pathway is considered a significant potential strategy for UPS therapy (36). Although the patient reported here underwent R0 resection and adjuvant chemotherapy, she experienced rapid recurrence, underscoring the critical importance of multimodal management in gastric UPS.

Nevertheless, certain aspects of the diagnostic and therapeutic process in this case warrant reflection. First, gastroscopy revealed a mucosal protuberance in the gastric fundus, and subsequent abdominal CT led to a preoperative diagnosis of GIST with an indication for surgery. Therefore, further tumor biopsy was not performed. Currently, Core biopsy to establish a definitive diagnosis is widely endorsed (37). Second, the follow-up period in this case was relatively short, with only the two-month postoperative outcome reported, and the patient’s long-term survival status remains unclear.

In conclusion, this case report and literature review underscore both the diagnostic challenges and the therapeutic limitations in managing gastric UPS. More effective and comprehensive therapeutic strategies are needed to improve patient survival.

## Patient perspective

Prior to admission, the patient experienced anorexia and speculated that it might be caused by the gastric mass. During the treatment process, she and her family were fully informed of the diagnostic and therapeutic challenges of UPS and the possibility of a poor prognosis, and they signed informed consent for surgery and chemotherapy. Postoperatively, the patient and her family expressed understanding of the highly aggressive nature of UPS. Despite recurrence two months after surgery, they remained actively engaged in follow-up care and expressed gratitude to the Gastric Cancer Center of West China Hospital, Sichuan University, for their efforts.

## Data Availability

The original contributions presented in the study are included in the article/**Supplementary Material**. Further inquiries can be directed to the corresponding author/s.

## Supplementary material

Supplementary Figure 1 Chest CT image showing a 0.3 cm nodule (red arrow) in the left lower lobe, bronchiectasis in the lower lobes bilaterally, minimal bilateral chronic inflammatory changes, and slight aortic wall calcification.

Supplementary Figure 2 Histopathological features of the gastric mass (Hematoxylin and eosin stain). **(A)** Low-power view (×200); **(B)** High-power view (×400).
