# Supplementary figures and images for: Primary undifferentiated pleomorphic sarcoma of the stomach mimicking a gastrointestinal stromal tumor: a case report and literature review
Source: Front Oncol. 2026 Apr 27;16:1777324. doi: 10.3389/fonc.2026.1777324 (PMC13158067; doi:10.3389/fonc.2026.1777324)

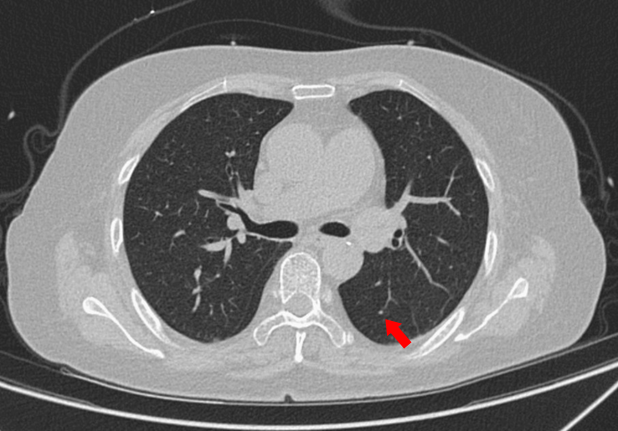

Supplement: Supplementary Figure 1 — Chest CT image showing a 0.3 cm nodule (red arrow) in the left lower lobe, bronchiectasis in the lower lobes bilaterally, minimal bilateral chronic inflammatory changes, and slight aortic wall calcification. [file Image1.jpeg]

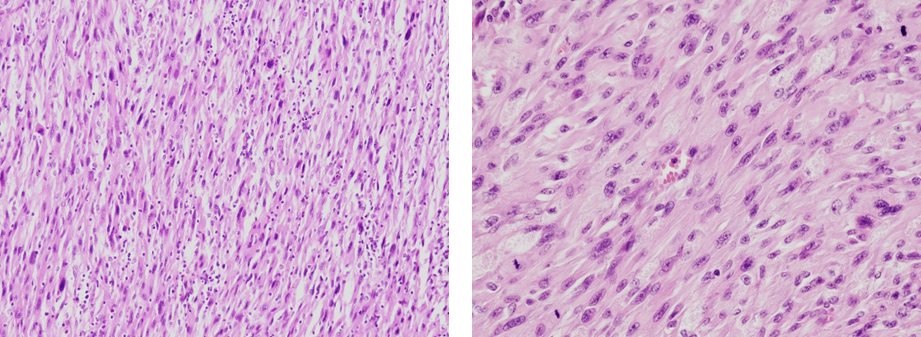

Supplement: Supplementary Figure 2 — Histopathological features of the gastric mass (Hematoxylin and eosin stain). (A) Low-power view (×200); (B) High-power view (×400). [file Image2.jpeg]
